# Isolation and Characterization of Primary Rat Valve Interstitial Cells: A New Model to Study Aortic Valve Calcification

**DOI:** 10.3791/56126

**Published:** 2017-11-20

**Authors:** Cui Lin, Dongxing Zhu, Greg Markby, Brendan M. Corcoran, Colin Farquharson, Vicky E. Macrae

**Affiliations:** ^1^Developmental Biology, The Roslin Institute and R(D)SVS, University of Edinburgh; ^2^Guangzhou Institute of Cardiovascular Disease, The Second Affiliated Hospital, School of Basic Medical Sciences, Guangzhou Medical University; ^3^Clinical Sciences and R(D)SVS, University of Edinburgh

**Keywords:** Developmental Biology, Issue 129, Calcific aortic valve disease, aortic valve interstitial cells, mineralization, calcium, phosphate, aorta

## Abstract

Calcific aortic valve disease (CAVD) is characterized by the progressive thickening of the aortic valve leaflets. It is a condition frequently found in the elderly and end-stage renal disease (ESRD) patients, who commonly suffer from hyperphosphatemia and hypercalcemia. At present, there are no medication therapies that can stop its progression. The mechanisms that underlie this pathological process remain unclear. The aortic valve leaflet is composed of a thin layer of valve endothelial cells (VECs) on the outer surfaces of the aortic cusps, with valve interstitial cells (VICs) sandwiched between the VECs. The use of a rat model enables the *in vitro *study of ectopic calcification based on the *in vivo *physiopathological serum phosphate (Pi) and calcium (Ca) levels of patients who suffer from hyperphosphatemia and hypercalcemia. The described protocol details the isolation of a pure rat VIC population as shown by the expression of VIC markers: alpha-smooth muscle actin (α-SMA) vimentin and tissue growth factor beta (TGFβ) 1 and 2, and the absence of cluster of differentiation (CD) 31, a VEC marker. By expanding these VICs, biochemical, genetic, and imaging studies can be performed to study and unravel the key mediators underpinning CAVD.

**Figure:**
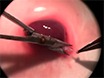


## Introduction

The healthy aortic valve is comprised of three leaflets, to which the distribution of mechanical stress during the opening and closing of the valve is equally apportioned. The valve leaflet has a defined structure of three distinct layers: the fibrosa, spongiosa, and ventricularis, which house valve interstitial cells (VICs) as the predominant cell type. These three layers are sandwiched between two beds of valve endothelial cells (VECs)^1^.

VICs play a critical role in the progression of Calcific Aortic Valve Calcification (CAVD), the most common heart valve disease in the Western world. CAVD is described as a progressive condition that is actively regulated by the valvular tissue and its surrounding microenvironment. Cellular changes initially cause fibrotic thickening, and eventually an extensive calcification of the aortic valve leaflets. This then leads to significant aortic valve stenosis and ultimately, left ventricular outflow obstruction^2^, leaving surgical valve replacement as the only viable treatment.

The pathophysiology of CAVD is complex, but shares similar mechanisms to physiological bone mineralization^3^. Whilst, a number of studies have demonstrated the ability of VICs to undergo osteogenic trans-differentiation and calcification^4^,^5^, the mechanisms underpinning this process have yet to be fully elucidated, highlighting the crucial requirement for a feasible and relevant *in vitro* model of CAVD.

Previous work by a number of laboratories has successfully isolated VICs from porcine and bovine models, and cultured these cells under calcifying conditions^6^,^7^,^8^. Due to the large size of the aortic valve in these models, isolation of cells through enzymatic digestion has been highly effective in generating pure populations of cells. However, these models can be restrictive due to the limited availability of molecular tools for large animal species. In contrast, rodent models remain advantageous due to relatively lower costs, potential for genetic manipulation, and the extensive array of molecular tools that are readily available. However, the isolation of VICs from small animal models is not widely employed, which is a likely consequence of the difficulties encountered when working with small tissue samples.

This detailed protocol reports a comprehensive method for the direct isolation of rat VICs. By careful dissection of the valve, followed by a series of enzymatic digestions, VICs can be isolated and employed in a wide variety of experimental techniques, including cell culture, calcification, and gene expression. This highly relevant *in vitro* model of CAVD will undoubtedly make an essential contribution to increasing our knowledge of this pathological process.

## Protocol

All animal experiments were approved by The Roslin Institute's Animal Users Committee, and the animals were maintained in accordance with Home Office (UK) guidelines for the care and use of animals. For the protocol described below, 5-week old, male Sprague Dawley rats were used.

### 1. Reagent Recipes

1. Prepare the **culture medium** using Dulbecco's Modified Eagle Medium (DMEM) and nutrient mixture F-12 (DMEM/F12). Add 10% sterilized heat-inactivated fetal bovine serum (FBS) and 1% gentamicin.
2. Prepare the **calcification medium** using culture medium and 2.7 mM Ca/2.5 mM Pi.
  1. Prepare **1 M calcium chloride** (CaCl_2_) by weighing out 555 mg of CaCl_2 _and dissolving it in 5 mL distilled water to make 5 mL of 1 M CaCl_2_. Filter the solution through a 0.22 µm syringe filter to sterilize the solution.
  2. Prepare **1 M sodium phosphate** by weighing out 710 mg anhydrous dibasic sodium phosphate (Na_2_HPO_4_) and 600 mg of anhydrous monobasic sodium phosphate (NaH_2_PO_4_), dissolving each separately in 5 mL distilled water. Combine 3,870 µL of Na_2_HPO_4 _and 1,130 µL of NaH_2_PO_4_, and filter through a 0.22 µm syringe filter to sterilize solution.
3. Prepare the **wash buffer** containing Hank's Balanced Salt Solution (HBSS) and 1% gentamicin.

### 2. Preparation of the Dissection Hood

1. Carry out all dissections in a ventilated hood, previously disinfected with 70% ethanol to ensure sterility of samples and reagents.
2. Sterilize dissection tools by autoclaving them followed by immersing the tips of the tools in a beaker containing 70% ethanol prior to use.
3. Prepare beakers containing wash buffer, and soak the dissection tools in wash buffer before coming into contact with the animal or tissues. Keep the wash buffer on ice at all times.

### 3. Extraction of Primary Rat VICs

NOTE: For the protocol described below, 5-week old, male Sprague Dawley rats were used.

1. Cull the rats (~ 100 g) by cervical dislocation in accordance with UK Home Office guidelines.
2. To dissect out the heart of each rat, place the animal supine on a glass dissection board, and disinfect the skin by spraying with 70% ethanol.
3. Make a 4 cm incision in the midline of the rat with the aid of dissection scissors, to expose the abdominal cavity, and carefully remove the rib cage and lungs, to expose the heart.
4. Remove the heart with a pair of sharp, spring curved scissors, and store the dissected heart in ice cold wash buffer until all gross dissections of the rats are complete.
5. To micro-dissect each heart, transfer the latter into a Petri dish covered in wash buffer. Trim the cardiac muscle with a pair of spring straight scissors (6 mm blade) to be left with a small area surrounding the ascending aorta and the aortic root.
6. Using the same spring straight scissors (6 mm blade), carefully cut open the ascending aorta towards the left ventricle and expose the aortic valve leaflets.
7. Transfer the opened aorta into a fresh, sterile Petri dish filled with HBSS. Dissect out the aortic valve leaflets, marked by their unique 'U' shape at the base of the aorta, with a pair of Vannas-type capsulotomy micro-scissors (3 mm blade).
8. Store all leaflets in 1 mL of ice cold wash buffer, in a 1.5 mL microcentrifuge tube until all dissections are complete.
9. Once all leaflets have been harvested, centrifuge them at 100 x g for 1 min at 4 °C to remove the wash buffer.
10. Perform subsequent steps in a cell culture hood to ensure sterilization. To remove the VECs, digest the leaflets in 100 µL 425 U/mL collagenase II for 5 min at 37 °C. Disrupt the digestion by gently pipetting up and down using a 200 µL pipette tip.
11. Centrifuge at 100 x g for 30 s to pellet the leaflets, and discard the supernatant carefully. Wash twice with 500 µL wash buffer and re-pellet the cells by centrifugation at 100 x g for 30 s.
12. To harvest the VICs from the leaflets, digest with 100 µL of 425 U/mL collagenase II for 2 h, and then release the VICs by gently pipetting up and down using a 200 µL pipette tip.
13. Dilute the collagenase II in 19 mL of culture medium and centrifuge at 670 x g for 5 min to pellet the VICs and remaining valve leaflet debris. Discard supernatant, and transfer leaflets and VICs to culture plates/flasks accordingly (**Table 1**).
14. Culture the VICs for 5-7 days in culture medium, until confluency is reached at 37 °C, in the presence of 5% (carbon dioxide) CO_2_, changing the medium after 72 h. For subsequent use in *in vitro *experiments, passage up to 5 times once confluency reaches 100%.

### 4. Induction of Calcification of Rat VICs

NOTE: For all experiments, count cells with a hemocytometer.

1. Perform all primary cell seeding and passaging in sterilized hoods to prevent contamination. To prepare primary rat VICs for *in vitro *calcification experiments, seed the cells at a density of 150,000 cells/well in 6-well plates. Maintain in culture medium until ≥ 90% confluency (typically 72 h).
2. Treat the VICs with calcification versus control medium and incubate at 37 °C, in the presence of 5% CO_2_, for an additional 72 h.
3. To study the calcified primary rat VICs for subsequent downstream analyses, remove the calcification/control medium and wash the monolayers with wash buffer to remove non-bound Ca and Pi ions.

### 5. Rat VIC Characterization

1. For immunostaining to monitor for phenotypic markers such as vimentin and α-SMA, seed the rat VICs at a density of 150,000 cells/well in 6 well-plates containing cover slips (cells will grow on the surface of the cover slips), and leave to grow until 50% confluency.
2. Aspirate the culture medium and fix the cell monolayers with 4% paraformaldehyde (PFA) for 10 min before washing them 3 times with phosphate buffered saline (PBS), for 5 min each time. Caution: PFA is toxic and must be handled carefully.
3. Incubate the cell monolayer with blocking and permeabilization buffer (1x PBS, 5% of normal serum from the same species as the secondary antibody, 0.3% Triton X-100) for 1 h at room temperature.
4. Incubate the cell monolayers with mouse anti-vimentin and rabbit anti-α-SMA antibodies diluted in antibody dilution buffer (1% bovine serum albumin + 0.3% Triton X-100 in PBS) overnight at 4 °C, gently shaking on a rocker. NOTE: Negative controls consisted of non-conjugated mouse IgG and rabbit IgG, using the same dilutions as the test samples.
5. On the next day, wash off the primary antibodies 3 times with PBS, for 5 min each time.
6. Incubate the cell monolayers with fluorophore-conjugated secondary antibodies for 1 h at room temperature, gently shaking on a rocker.
7. Wash 3 times with PBS, and gently tweeze out the coverslips (which contain rat VICs) with a pair of forceps and place on a coverslip containing 4',6-diamidino-2-phenylindole (DAPI). Leave the slides to cure for at least 24 h at 4 °C before visualizing with a fluorescence microscope.
8. For Western blot analysis, seed the rat VICs at a density of 150,000 cells/well in 6 well-plates and leave to grow in culture medium until 100% confluent.
9. Use 8 µg of protein to run a Western blot to measure the expression of CD31 and rule out any VEC contamination. NOTE: A standard Western blot protocol was followed as described previously^9^.
10. For gene studies, extract ribonucleic acid (RNA) using a commercial kit following the manufacturer's guidelines.
11. Obtain cDNA using reverse transcription to measure target genes' expression using polymerase chain reaction (PCR) and real-time PCR (RT-PCR; also known as qPCR) with green fluorophore detection, using *Gapdh* as the reference gene, as previously described^10^.
  1. Use the following program for PCR analysis: 1 cycle of 94 °C for 3 min, 30 cycles of 94 °C for 30 s, 63 °C for 30 s, 72 °C for 35 s, and finally 1 cycle of 72 °C for 5 min.
  2. Use the following program for qPCR analysis: 1 cycle of 95 °C for 10 min, 40 cycles of 95 °C for 15 s, 60 °C for 1 min and an additional cycle of 95 °C for 1 min, 55 °C for 30 s, and 95 °C for 30 s.
12. Run a gel electrophoresis to analyze PCR products using a 2% agarose gel in Tris/Borate/EDTA (TBE) buffer.

### 6. Rat VIC Calcification Studies

1. For Alizarin Red S and biochemical calcification studies, please follow seeding and calcification guidelines described in section 4.
2. Stain the cell monolayers with 5% Alizarin red S solution, gently rocking on a shaker, for 20 min. Subsequently wash 3 times with distilled water, for 5 min each time. Acquire images of each well.
3. To quantify Ca deposition, use a biochemical Ca assay kit. Leach Ca^2+^ ions using 0.6 M hydrochloric acid (HCl) for 24 h at 4 °C, with gentle agitation.
4. Harvest the supernatant and measure the Ca concentration using a Ca assay kit (see **Table of Materials**) following the manufacturer's guidelines.
5. Calculate the calcium concentration as a fraction of total cellular protein. Denature cellular proteins from the cell monolayers with 0.1 M sodium hydroxide (NaOH) + 0.1% sodium dodecyl sulphate (SDS). Determine the total protein concentration using a detergent compatible (DC) protein assay following the manufacturer's guidelines.

## Representative Results

The aim of this protocol was to describe the isolation of primary rat VICs and culture them for *in vitro* calcification experiments. By employing the method described above, rat VICs can be successfully isolated and expanded for the study of the mechanisms responsible for CAVD.

### Rat Primary VICs Co-localize with Established VIC Markers:

The VIC phenotype of isolated cells was confirmed through immunofluorescence by probing for the VIC markers: vimentin and α-SMA (red and green, respectively, Figure 1A), and is in agreement with previous reports^11^,^12^. The representative negative controls using non-conjugated mouse and rabbit IgG are shown in Figure 1B. Additionally, the expression of the VIC-growth regulator TGFβ-1 and TGFβ-2 was confirmed using PCR analysis (Figure 1C). In order to confirm that the isolated rat primary VICs were free from endothelial contamination, Western blot analysis was performed to verify that rat VICs were negative for the endothelial cell marker, CD31, using canine mitral VECs as a positive control (Figure 1D).

### Ca and Pi Induce Rat VIC Calcification:

Elevated systemic Ca and/or Pi concentrations typically drive the calcification of VICs *in vitro*. To appreciate the calcification potential of the isolated rat VICs, the cells were exposed to elevated levels of Ca and Pi, which mimic pathological hypercalcemia and hyperphosphatemia conditions in ESRD patients. Treatment of VICs with 2.7 mM Ca/2.5 mM Pi induced calcification, as determined by Alizarin Red S staining for Ca deposition (Figure 2A) and colorimetric determination of Ca levels following HCl leaching (81 fold; *p *< 0.05 using Student's *t*-Test; Figure 2B).

### Gene Expression Changes Associated with VIC Calcification:

The calcification of vascular cells *in vitro* is associated with a distinct molecular profile. In the present study, the treatment of VICs with 2.7 mM Ca/2.5 mM Pi induced a significant increase in the mRNA expression of the osteogenic markers: Msh homeobox 2, *Msx2* (2.04 fold change; *p *< 0.01; Figure 3A), alkaline phosphatase, *Alpl* (1.49 fold change; *p *< 0.001; Figure 3B), and phosphoethanolamine/phosphocholine phosphatase, *Phospho1* (4.7 fold change; *p *< 0.01 using one-way ANOVA; Figure 3C). However, the expression of the osteogenic marker, *Runx2*, and calcification inhibitor ectonucleotide pyrophasphatase, *Enpp1*, remained unchanged (Figure 3D**-E**).

**Figure:**
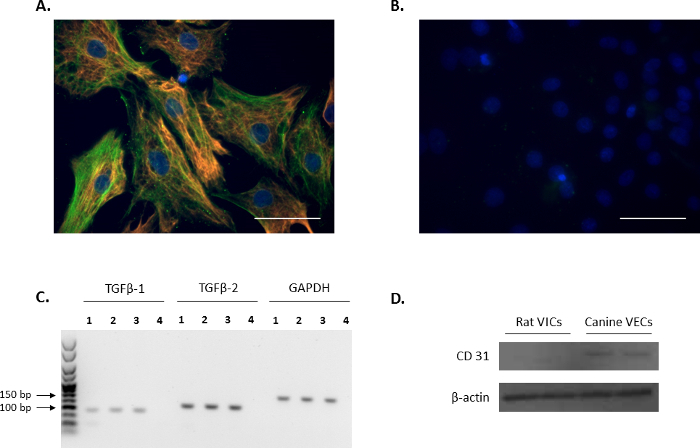

**Figure 1. Expression of VIC markers.** (**A**) Immunofluorescence showing double staining and colocalization of alpha-smooth muscle actin (α-SMA; green) and vimentin in valve interstitial cells (VICs). (**B**) Representative image of negative controls using mouse and rabbit IgG. Nuclei are stained in blue using 4',6-diamidino-2-phenylindole (DAPI). Scale bar = 50 µm. (**C**) The presence of transforming growth factor beta 1 (*TGFβ-1*) and *TGFβ-2* in VICs (Lanes 1-3) as shown by PCR analysis. Lane 4 is the water control. Reference gene used was *Gapdh*. (**D**) Western blot analysis showing the abundant expression of CD31 in canine mitral valve endothelial cells (VECs) in comparison to no expression in VICs. Please click here to view a larger version of this figure.

**Figure:**
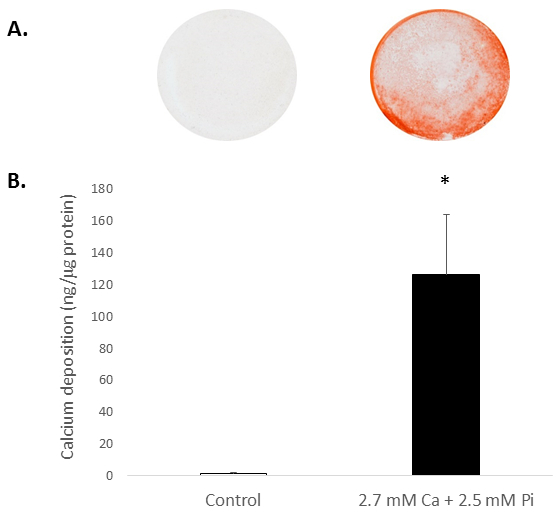

**Figure 2. *In vitro* calcification of rat VICs.** Ca deposition in VICs treated with 2.7 mM Ca/2.5 mM Pi as determined by: (**A**) Photograph showing Alizarin Red S staining of whole cell monolayers in the well, and (**B**) colorimetric determination of Ca levels following HCl leaching. Student's *t*-test was performed to analyze the significance between the two data groups. Results are presented as mean ± S.E.M. *p < 0.5 compared with control; (n = 4).

**Figure:**
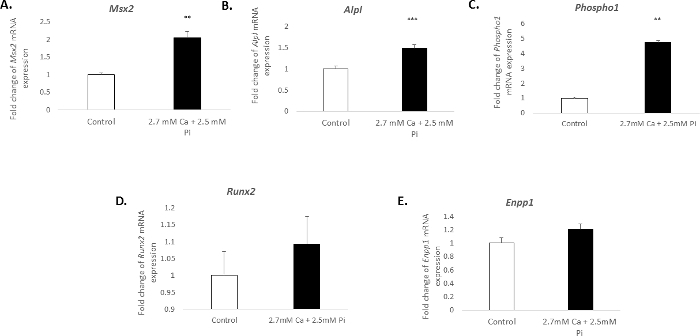

**Figure 3. Gene expression changes associated with VIC calcification.** Fold change in the mRNA expression of osteogenic markers (**A**) *Msx2*, (**B**) *Alpl*, (**C**) *Phospho1*, (**D**) *Runx2*, and (**E**) *Enpp1* in VICs treated with 2.7 mM Ca/2.5 mM Pi for 48 h. mRNA expression is shown as a fold change compared to the endogenous reference gene *Gadph.* One-way ANOVA using general linear model incorporating pair-wise comparisons was performed to analyze the significance between multiple groups. Results are presented as mean ± S.E.M. **p < 0.01; ***p < 0.001 compared to control; (n = 6). Please click here to view a larger version of this figure.

**Table:** 

**Number of valve leaflets**	**Culture plate/flask**
9 to 15	1 well in 12-well plate
15 to 30	1 well in 6-well plate
30+	T25

**Table 1. **General guidelines for the number of leaflets required for initial seeding.

## Discussion

This detailed protocol describes a practical method for the acquisition of primary rat VICs, enabling the isolation of these cells from rat heart valves through enzymatic digestion. Our method further supports and extends the use of a previously reported rat *in vitro* model to study aortic valve calcification^13^. The isolation of VICs from the aortic valve introduces the potential for contamination from neighboring VECs. However, our immunofluorescence data confirm that the initial digestion step is sufficient to remove the VECs, rendering the isolated cells negative for the endothelial cell marker, CD31. Furthermore, α-SMA staining confirms the activated phenotype of the VICs, which is required for calcification^12^.

VIC isolations have previously been reported in large animal models^6^,^7^,^8^. However, these species are constrained by the limited genetic and molecular tools available for downstream study, as well as their restricted accessibility in laboratories around the world. In contrast, such tools are well established in readily available rodents and therefore, the ability to isolate rat-derived VICs enables a greater experimental design capacity. The use of VICs from young rats also means that the cells are relatively more proliferative than older rats, therefore requiring less animals to yield more cells. Although mice are readily accessible, but due to mice having notably smaller hearts, the isolation of primary mouse VICs would be more time-consuming and considerably more animals would be required to isolate the same yield of cells as the rat model.

A significant advantage of this described approach is that VICs can be isolated temporally from wild type and transgenic rats, as well as rat models of cardiovascular disease and valve injury. Using the rat model would require a larger number of animals for large scale experiments, therefore to reduce animal use, primary rat VICs can be transformed to produce a cell line once it has been well characterized.

Whilst the severe clinical implications of CAVD are widely recognized, the underlying causative mechanisms have yet to be determined. In addition, effective medication therapies that may prevent and potentially cure aortic valve calcification are not available at present. The culture of VICs under calcifying conditions therefore provides a highly relevant *in vitro* model of CAVD. We show that elevated Ca and Pi levels induce the *in vitro* calcification of isolated rat VICs with a concomitant increase in the expression of osteogenic gene markers *Msx2*, *Alpl*, and *Phospho1*. It is widely established that these markers are associated with the pathological process of vascular calcification^14^,^15^,^16^. Our data therefore show that the culture of rat VICs in calcifying medium is an appropriate model with which to study aortic valve calcification *in vitro*. Indeed, recent studies from our laboratory have utilized this model to demonstrate that Ca promotes aortic valve calcification through Annexin VI enrichment of VIC-derived matrix vesicles^17^.

Despite the benefits of using rat VICs and their efficient characterization, some limitations still exist. First, the size of VIC population within rat valves is very small, and therefore many animals are needed in order to generate sufficient cell numbers for extensive *in vitro* studies. However, it is possible to overcome this limitation by undertaking preliminary studies in immortalized valve interstitial cell lines, as recently reported by us^18^, and subsequently employing primary cultures to verify and extend these findings.

In summary, the described method explains the successful isolation, culture, and calcification of primary rat VICs, which may be subsequently assessed using a variety of analyses including biochemical assays, as well as protein and RNA analyses. This model offers a reliable and convenient system in which to investigate CAVD *in vitro*, and provides a valuable tool for investigating the molecular mechanisms responsible for this destructive disease.

## Disclosures

The authors have nothing to disclose.
